# Effects of Long-term Cotton Continuous Cropping on Soil Microbiome

**DOI:** 10.1038/s41598-019-54771-1

**Published:** 2019-12-04

**Authors:** Hui Xi, Jili Shen, Zheng Qu, Dingyi Yang, Shiming Liu, Xinhui Nie, Longfu Zhu

**Affiliations:** 1 College of Agronomy, Shihezi University, Shihezi, 832000 P.R. China; 2 State Key Laboratory of Agricultural Microbiology, Huazhong Agricultural University, Wuhan, 430070 Hubei P.R. China; 3 National Key Laboratory of Crop Genetic Improvement, Huazhong Agricultural University, Wuhan, 430070 Hubei P.R. China

**Keywords:** Microbiology, Plant sciences

## Abstract

Verticillium wilt is a severe disease of cotton crops in Xinjiang and affecting yields and quality, due to the continuous cotton cropping in the past decades. The relationship between continuous cropping and the changes induced on soil microbiome remains unclear to date. In this study, the culture types of 15 isolates from Bole (5F), Kuitun (7F), and Shihezi (8F) of north Xinjiang were sclerotium type. Only isolates from field 5F belonged to nondefoliating pathotype, the others belonged to defoliating pathotype. The isolates showed pathogenicity differentiation in cotton. Fungal and bacterial communities in soils had some difference in alpha-diversity, relative abundance, structure and taxonomic composition, but microbial groups showed similarity in the same habitat, despite different sampling sites. The fungal phyla Ascomycota, and the bacterial phyla Proteobacteria, Actinobacteria, Chloroflexi, Acidobacteria and Gemmatimonadetes were strongly enriched. *Verticillium* abundance was significantly and positively correlated with AN, but negatively correlated with soil OM, AK and pH. Moreover, *Verticillium* was correlated in abundances with 5 fungal and 6 bacterial genera. Overall, we demonstrate that soil microbiome communities have similar responses to long-term continuous cotton cropping, providing new insights into the effects of continuous cotton cropping on soil microbial communities.

## Introduction

Continuous cropping regimes can cause crop yield reduction, soil-borne plant pathogen accumulation^1–3^, and soil microbial community disruption^4–6^. In spite of these negative feedbacks, this regime is still common in many agricultural production systems. Cotton is an important economic crop of China, mainly cultivated in Xinjiang, where long-term continuous cropping results in lower yields, also caused by a severe incidence of Verticillium wilt disease, which hinders the development of the national cotton industry.

Verticillium wilt is a catastrophic disease, caused by *Verticillium dahliae*^7^. Soil-borne disease are considered as influenced also by changes affecting the below ground microbial communities, a situation summarized by the term “microbiome disease”^8^. If bacteria, fungi, and nematode pathogens act with a synergistic effect, it may become very difficult to control root colonization and insurgence of such a disastrous disease^9,10^. Under the cotton continuous cropping system, the increase in prevalence of soil-borne diseases is directly linked to the increase of the pathogen abundance^4^, and the occurrence of different *V*. *dahliae* pathotypes^11^. Due to the variety of physiological types of *V*. *dahliae*, understanding how they affect the soil microbial community among different regions may be useful for an effective disease management.

The soil microbiome provides several services, including organic matter dynamics, nutrient cycling and suppression or regulation of soil-borne diseases^12^, underpinning soil quality and function in the agroecosystem^13^. In recent years, the important role of soil microbiome in regulating agricultural production has been elucidated. Upon infection, plants can recruit microbes that promote disease resistance and plant growth with an effect on the composition of the root microbiome^14^. For example, a rhizosphere *Flavobacterium* sp. was found to enhance wilt resistance in tomato^15^. A number of links are active among diseased plants and the community composition and function of the soil microbiome^16–18^. However, the majority studies have focused on either bacterial or fungal communities alone.

Compared to cultivated soil, the non-rhizosphere one is capable to keep soil aggregates stable sustaining resistance to unfavorable environmental conditions such as drought^19^. In fact, non-rhizosphere soils have an important role for the next generation of crops. Few studies focused thus far on the effect of continuous cropping regimes on the non-rhizosphere or bulk soil microbiomes^20^.

In this study, we tested pathotype, growth rate, spore production and pathogenicity of 15 representative *V*. *dahliae* isolates, and analyzed fungal and bacterial community compositions of 15 bulk soils, from three cotton producing regions, using Ilumina MiSeq sequencing. The study aim was to (i) determine the diversity and pathogenic differentiation of *V*. *dahliae* from cotton in north Xinjiang; (ii) compare the structure and composition of fungal and bacterial communities in soils of three cotton fields; and (iii) to explore potential links between Verticillium wilt prevalence and soil properties or microbial communities.

## Results

### Pathotype, and pathogenic differentiation of *Verticillium dahliae* isolates

**Figure 1:**
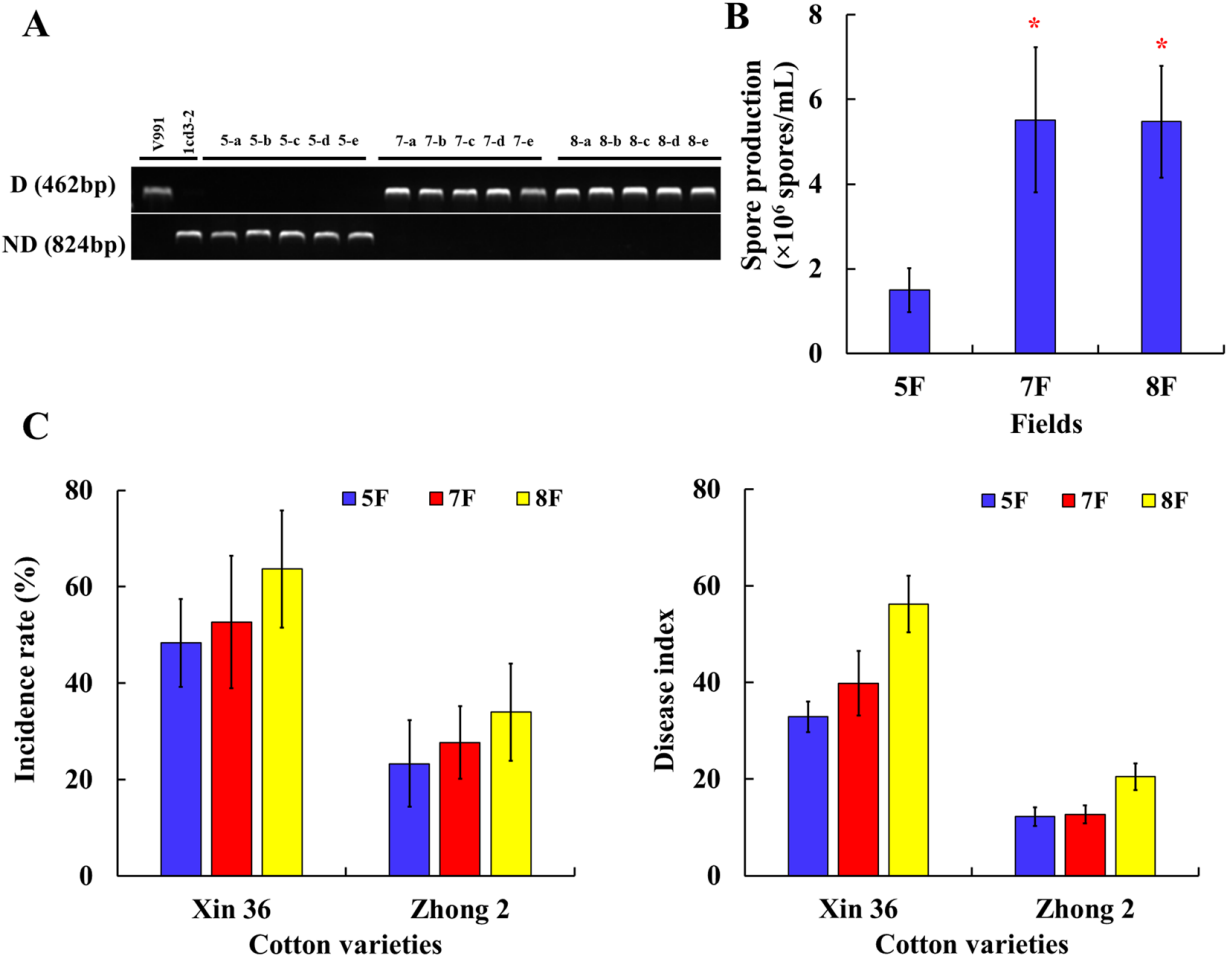
The results of the pathotypes, cultural characters and pathogencity of *Verticillium dahliae*. (**A**) Identification of the defoliating and non-defoliating pathotypes of all isolates with the corresponding molecular marker (see Fig. S1 for the full-length gels). (**B**) The spore production of 15 isolates. Red asterisk represents the spore production of D pathotype as significantly higher than that of ND pathotype. (5F vs 7F, P = 0.047; 5F vs 8F, P = 0.049; ^*^P < 0.05). (**C**) The results of pathogenic assay, including the statistics of the incidence rate and disease index.

**Figure 2:**
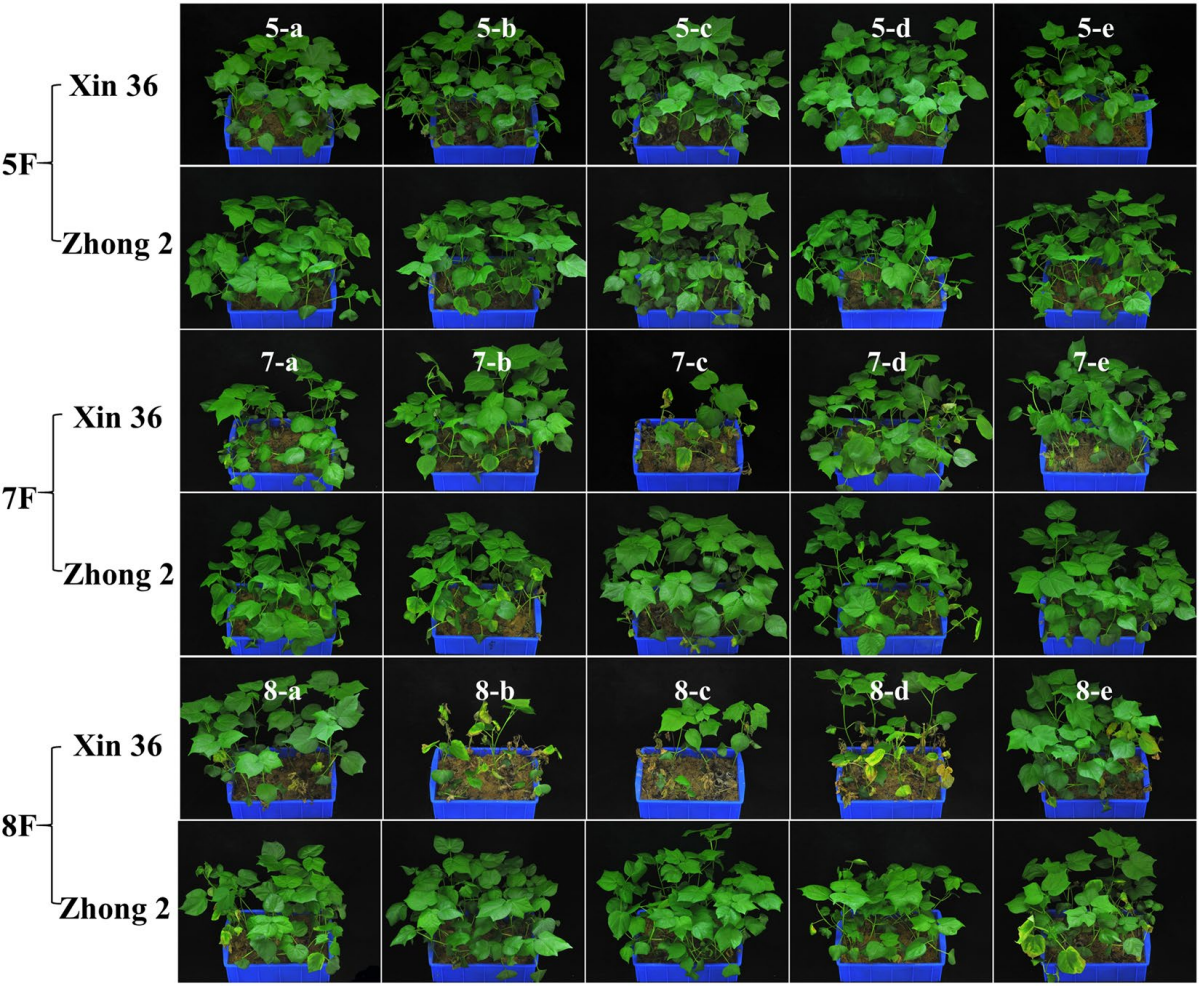
Phenotypes of two cotton varieties after inoculation with the 15 isolates tested. Each isolate was inoculated with 30 seedlings of two cotton varieties, respectively. Sterile distilled water was used as control. Replicated twice. When the disease index reached 50.0 after inoculation with V991 on Xinluzao 36, the disease indexes were counted.

The pathotypes of 15 *V*. *dahliae* isolates proceeding from the three cotton fields was determined via a specific PCR assay. Isolates from fields 7F and 8F belonged to the defoliating (D) pathotype (462 bp specific fragment), whereas those from field 5F belonged to the nondefoliating (ND) one (824 bp specific fragment) (Fig. 1A and S1). Morphological observations showed that all isolates were sclerotium type (Fig. S2). Their growth exhibited no significant difference among the three cotton fields (P = 0.783), and also between the D and ND pathotypes, whereas the spore production of D pathotype as significantly higher than that of ND pathotype (5F vs 7F, P = 0.047; 5F vs 8F, P = 0.049) (Fig. 1B). The results of the pathogenicity assay indicated different levels of disease resistance response in cotton varieties (Xinluzao 36, DI = 42.96, P = 0.029; Zhongzhimian 2, DI = 15.14, P = 0.038) vs the 15 isolates (Fig. 1C). The isolates had evident pathogenicity differentiations on the two varieties (Fig. 2), included the two reference strains (Fig. S3). The pathogenicity of the isolates from the three cotton fields were 8F > 7F > 5F (Table. S1). Moreover, the growth rate of all isolates showed no correlation with disease index, however, spore production (R = 0.534*, P = 0.027) showed a significant and positive correlation with the average disease index (Fig. S4).

### Alpha-diversity of fungal and bacterial species in soils under long-term continuous cotton cropping

To profile soil microbiome of the three cotton fields under continuous cropping regime in north Xinjiang, 15 soil samples were sequenced by Illumina MiSeq. A total of 1407,987 ITS1 and 484,724 V3-V4 16S rRNA high quality sequencing reads were analyzed. Coverages (> 99% for fungi; > 92% for bacteria) suggested that the identified sequences represented most fungi and bacteria present in the soil samples (Table 1). The high-quality sequences were gathered into 1344 fungal OTUs and 4717 bacterial OTUs at 97% sequence identity, respectively.

**Table 1:** Alpha-diversity indices of soil microbiomes among three cotton fields. (Values are the means ± standard deviation, n = 5).

	Sample fields	Coverage	OTUs	Shannon	Chao1	Evenness	Phylogenetic diversity
Fungal Community	5F	0.999 ± 0.0001	871	4.84 ± 0.09	524.45 ± 1.40	0.55 ± 0.01	98.98 ± 4.55
	7F	0.998 ± 0.0001	842	4.22 ± 0.09	568.37 ± 1.29	0.48 ± 0.01	86.78 ± 3.03
	8F	0.998 ± 0.0002	863	3.58 ± 0.26	531.44 ± 2.75	0.41 ± 0.03	88.68 ± 4.70
Bacterial Community	5F	0.923 ± 0.0009	3812	9.41 ± 0.06	3379.91 ± 3.13	0.84 ± 0.005	135.49 ± 1.01
	7F	0.928 ± 0.0003	3657	9.52 ± 0.01	3194.69 ± 9.38	0.86 ± 0.001	132.98 ± 1.04
	8F	0.925 ± 0.0011	4717	9.43 ± 0.02	3309.12 ± 3.38	0.85 ± 0.002	129.61 ± 0.75

The diversity (Shannon), richness (Chao1), evenness and phylogenetic diversity indices of fungal and bacterial communities are shown in Table 1. There were significant differences in Shannon (P = 0.004) and evenness index (P = 0.005) of fungi, chao1 (P = 0.007), evenness index (P = 0.008) and phylogenetic diversity (P = 0.006) of bacteria among the three sample groups. Shannon (P = 0.032) index of bacteria showed significant differences among the soil samples of the three sample groups. Significantly higher diversity and evenness in 5F samples were found for fungi, with a lower score for bacteria.

### Soil microbial community structures of long-term continuous cotton cropping fields

**Figure 3:**
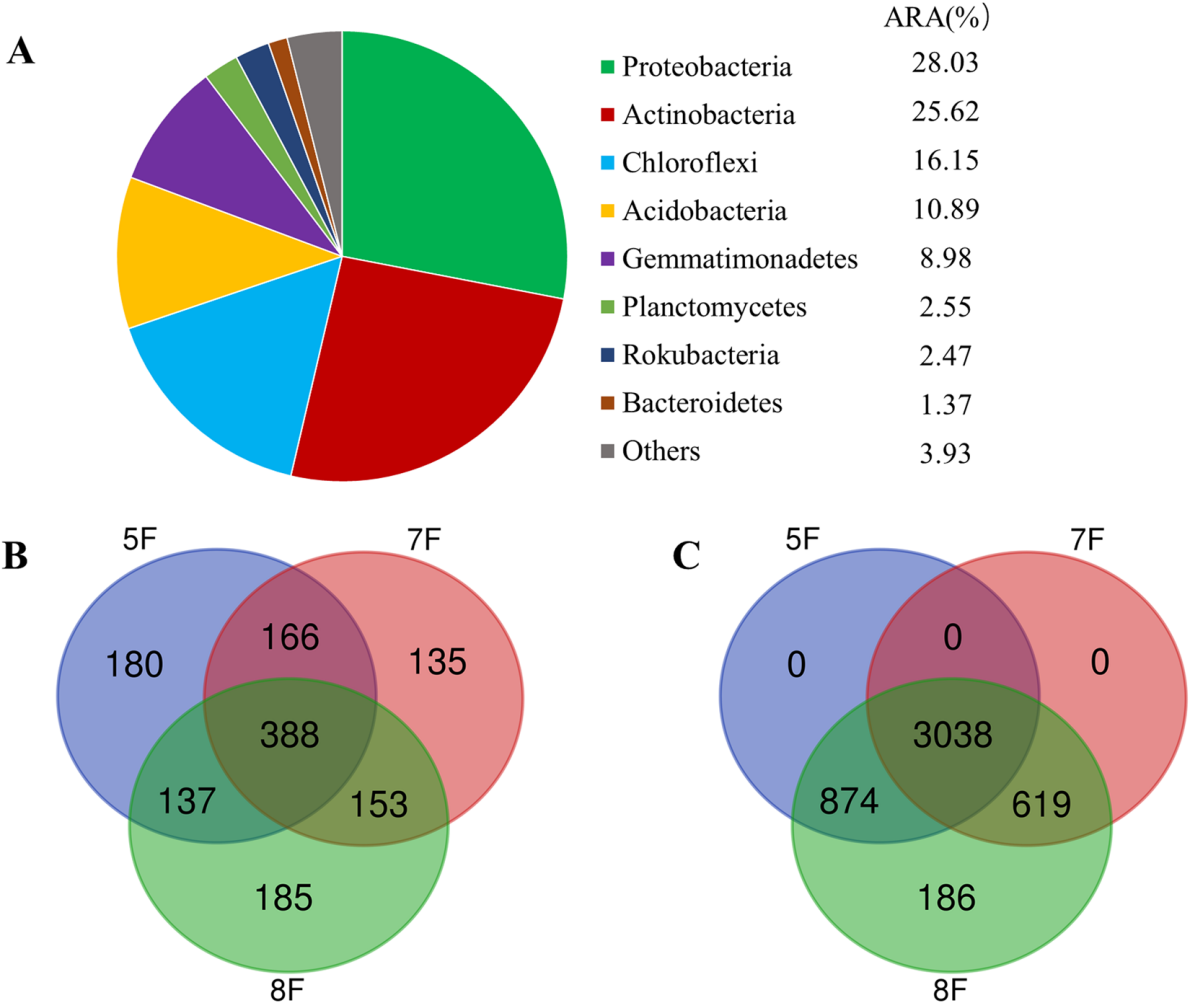
The microbiome compositions of bacterial (**A**) taxa at phylum level, and Venn diagrams of fungal (**B**) and bacterial (**C**) OTUs in soil samples from the three fields. (Five samples in each group, phyla with average relative abundance (ARA) < 1% were merged and indicated as “Others”).

According to 97% species similarity, 11 eukaryotic and 29 prokaryotic phyla were identified from fungal ITS and 16S rRNA gene sequences, respectively. Fungal OTUs were predominantly composed of phyla Ascomycota (93.04%), Basidiomycota (2.15%) and Mortierellomycota (1.71%). Bacterial OTUs mainly consisted of Proteobacteria (28.01%), Actinobacteria (25.60%), Chloroflexi (16.14%), Acidobacteria (10.89%), Gemmatimonadetes (8.98%), Planctomycetes (2.54%), Rokubacteria (2.47%) and Bacteroidetes (1.37%) (Fig. 3A).

Further investigation was conducted through Venn diagram to identify the dominant OTUs present in the three cotton fields. The fungal and bacterial OTUs shared among the three fields represented 28.87% and 64.41% of total reads, respectively (Fig. 3B,C). For fungi, 13.39%, 10.04% and 13.76% unique OTUs were found in 5F, 7F and 8F, respectively, whereas 3.94% unique OTUs were found only in field 8F for bacteria.

### Difference in soil microbial community compositions

**Figure 4:**
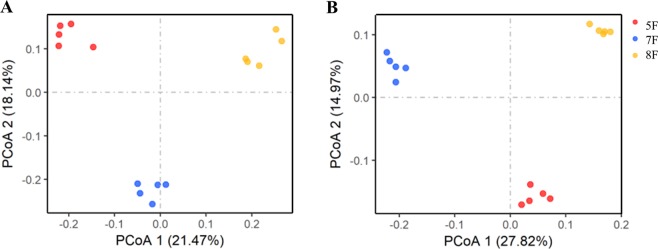
Unweighted unifrac- PCoA plots of fungal (**A**) and bacterial communities (**B**) at OTU level in three groups.

The soil microbiome compositions of different cotton fields at phylum level and UniFrac-unweighted principal coordinate analysis (PCoA) based on the OTU level were analyzed. In the 11 fungal and 29 bacterial phyla identified, 3 fungal and 8 bacterial phyla had a relative abundance above 1% in the three groups. At the phylum level, the dominant phyla (relative abundance above 5% at least in one sample) in three fields were Acomycota, Proteobacteria, Acitnobacteria, Chloroflexi, Acidobacteria and Gemmatimonadetes (Fig. 3A). PCoA analysis showed variations among the 15 soil samples for fungi and bacteria. Both fungal and bacterial communities from three cotton fields could be distinctly separated from each other (Fig. 4A,B).

**Figure 5:**
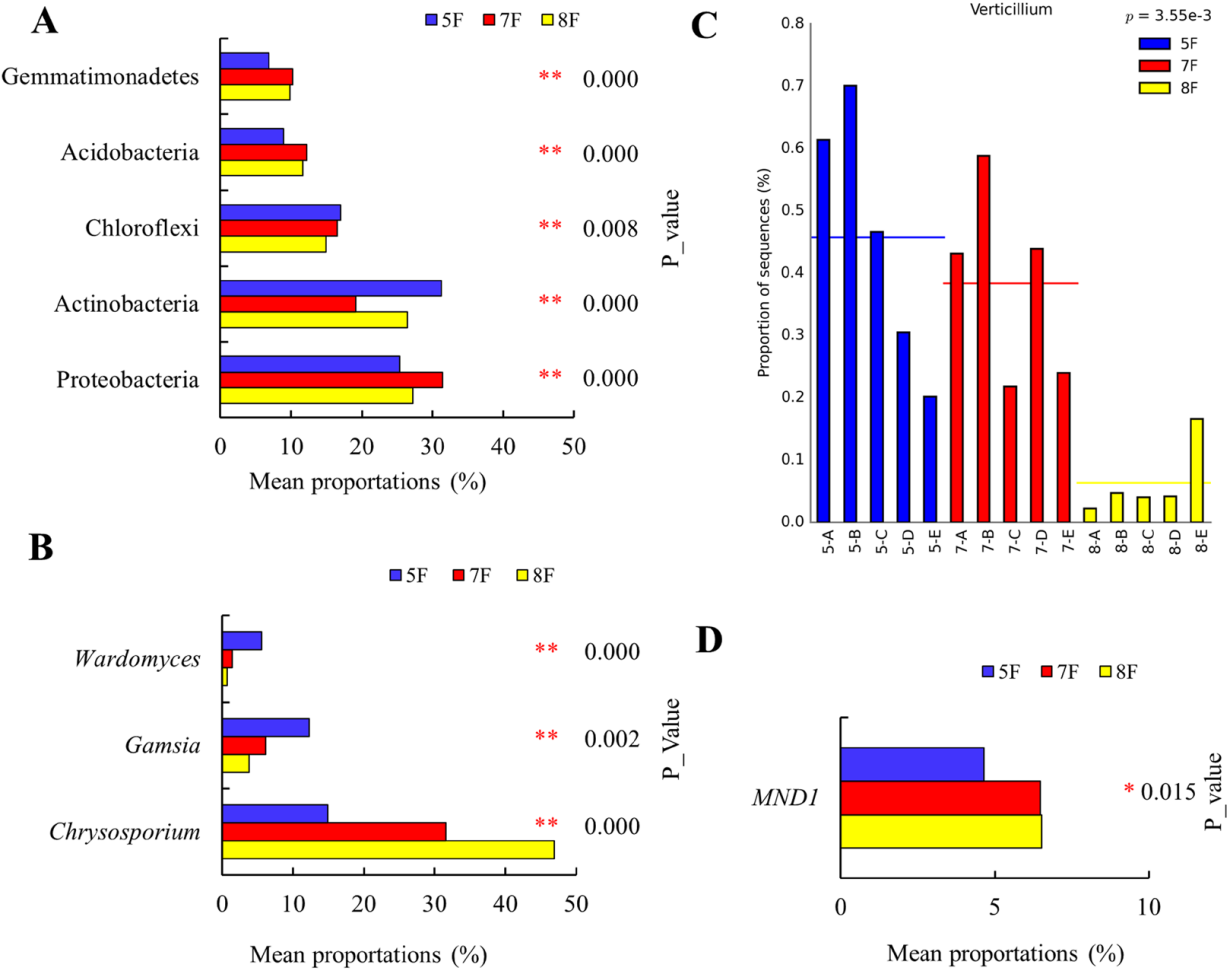
Comparison for abundance of fungal and bacterial sequences in the three sample groups. Bacterial (**A**) abundance at the phylum level. Fungal (**B**) and bacterial (**D**) abundances at the genus level. (**C**) *Verticillium* abundance in the three sample groups. (^*^P < 0.05; ^**^P < 0.01).

At the phylum and genus level, statistically significant differences were found in both fungal and bacterial taxa among three cotton fields. The dominant phylum in fungi was Ascomycota (5F = 91.13 ± 4.38%, 7F = 95.79 ± 0.51% and 8F = 92.19 ± 6.97%, P = 0.379), with no significant difference among the three fields. Dominant bacterial phyla were Proteobacteria (P = 0.000), Actinobacteria (P = 0.000), Chloroflexi (P = 0.008), Acidobacteria (P = 0.000) and Gemmatimonadetes (P = 0.000), which exhibited significant differences among the three sample groups (Fig. 5A).

253 fungal genera and 603 bacterial genera were identified, of which 47 (fungal) and 269 (bacterial) were differentially represented (P < 0.05). The dominant fungal genera were *Chrysosporium*, *Gamsia* and *Wardomyces*, with significant differential abundance among the three sample groups (Fig. 5B). Additionally, the relative abundance of *Verticillium* showed extremely statistically significant differences among the three groups (Fig. 5C). The dominant bacterial genera were an unidentified *Gemmatimonadetes* (5F = 6.61 ± 0.9%, 7F = 7.01 ± 0.51% and 8F = 10.11 ± 0.89%, P = 0.000), *MND1* and an unidentified member of Proteobacteria (5F = 2.55 ± 0.29%, 7F = 9.34 ± 2.27% and 8F = 1.00 ± 0.26%, P = 0.000) (Fig. 5D).

### Soil physicochemical properties and microbiome

**Figure 6:**
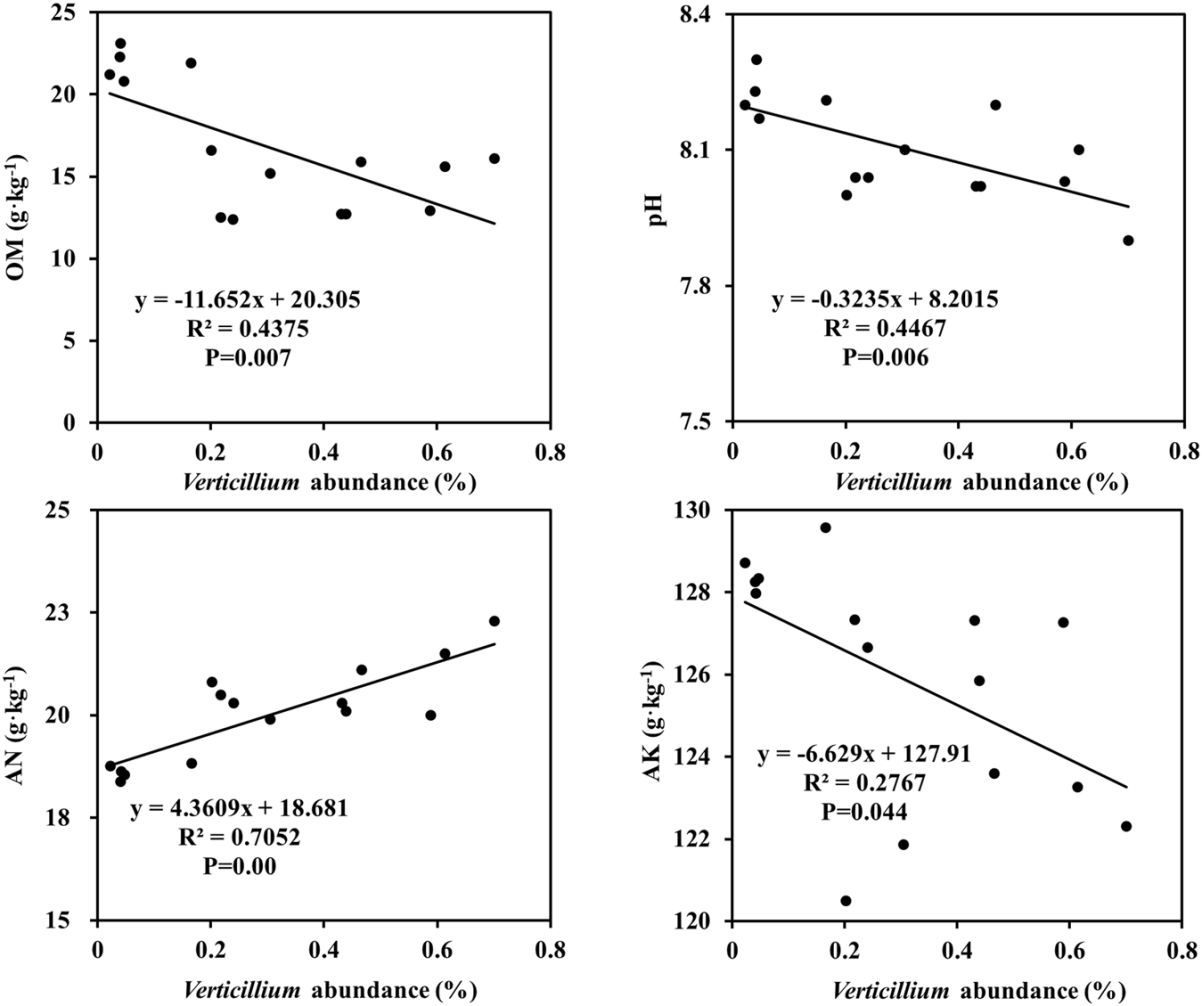
Correlations between *Verticillium* abundance and soil physicochemical properties (see Table 2 for variable acronyms).

In this study, all soil variables but total phosphorus (TP) exhibited significant differences among the three cotton fields (Table 2). The results of Mantel tests revealed that fungal and bacterial community structures were connected to soil physicochemical properties (Table 3). Pearson correlation coefficient also showed alpha diversity indices had some significant positive or negative correlations with some of the measured soil variables (Table 4). Fungal Shannon and evenness indices were only positively correlated with soil alkali-hydrolyzable nitrogen (AN), but negatively correlated with organic matter (OM), available potassium (AK) and pH. In contrast, bacterial Chao1 was positive correlated with EC and negatively correlated with available phosphorus (AP), whereas evenness showed a positive correlation with AP. Besides, bacterial phylogenetic diversity showed positive correlation with AN, and a negative correlation with OM, AK and pH. Meanwhile, *Verticillium* abundance was significantly and positively correlated with soil AN, but negatively correlated with OM, AK and pH (Fig. 6).

**Table 2:** Soil physicochemical properties of three cotton fields. (Values are means ± standard deviation. The same letter means no significant difference).

Cotton fields	Organic Matter (OM)	Alkali-hydrolyzable nitrogen (AN)	Available phosphorus (AP)	Available potassium (AK)	pH	Electrical conductivity (EC)	Total nitrogen (TN)	Total phosphorus (TP)
5F	15.88 ± 0.23b	21.12 ± 0.40a	7.41 ± 0.05c	122.30 ± 0.55c	8.06 ± 0.05b	0.15 ± 0.00a	0.04 ± 0.00b	0.03 ± 0.00a
7F	12.64 ± 0.09c	20.24 ± 0.09b	15.15 ± 0.01a	126.88 ± 0.29b	8.03 ± 0.00b	0.12 ± 0.00c	0.05 ± 0.00ab	0.02 ± 0.00b
8F	21.86 ± 0.41a	18.63 ± 0.08c	8.18 ± 0.02b	128.57 ± 0.28a	8.22 ± 0.02a	0.14 ± 0.00b	0.05 ± 0.00a	0.02 ± 0.00ab

**Table 3:** Correlations between soil physicochemical properties and fungal or bacterial community by Mantel test. (^*^P < 0.05; ^**^P < 0.0^1^ n = 15, see Table 2 for variable acronyms).

Soil variables	OM	AN	AP	AK	PH	EC	TN	TP
Fungal community	0.3492^**^	0.5591^**^	—	0.7904^**^	0.2746^*^	0.4593^**^	0.2303^*^	—
Bacterial community	0.7461^**^	0.3596^**^	0.8382^**^	0.3626^**^	0.4005^**^	0.5006^**^	—	—

**Table 4:** Correlations between soil physicochemical properties and fungal or bacterial community indices. (^*^ P < 0.05; ^** ^P < 0.0^1^ n = 15, see Table 2 for variable acronyms).

Soil variables	Fungi				Bacteria			
	Shannon	Richness	Evenness	Phylogenetic diversity	Shannon	Richness	Evenness	Phylogenetic diversity
OM	-0.582^*^	-0.343	-0.582^*^	-0.085	−0.315	0.325	-0.306	-0.560^*^
AN	0.693^**^	-0.122	0.717^**^	0.172	0.24	0.038	0.126	0.753^**^
AP	-0.071	0.447	−0.083	-0.368	0.509	-0.772^**^	0.630^*^	-0.007
AK	-0.780^**^	0.149	−0.799^**^	-0.505	0.193	-0.481	0.349	-0.713^**^
PH	-0.518^*^	-0.244	-0.529^*^	-0.044	-0.433	0.225	-0.367	-0.626^*^
EC	0.419	-0.386	0.446	0.345	-0.338	0.650^**^	-0.488	0.410
TN	-0.513	0.070	−0.505	-0.587^*^	0.127	-0.397	0.291	-0.563^*^
TP	0.395	0.094	0.388	0.535^*^	0.104	0.477	-0.098	0.346

### Correlations between *Verticillium* and other genera abundance

**Figure 7:**
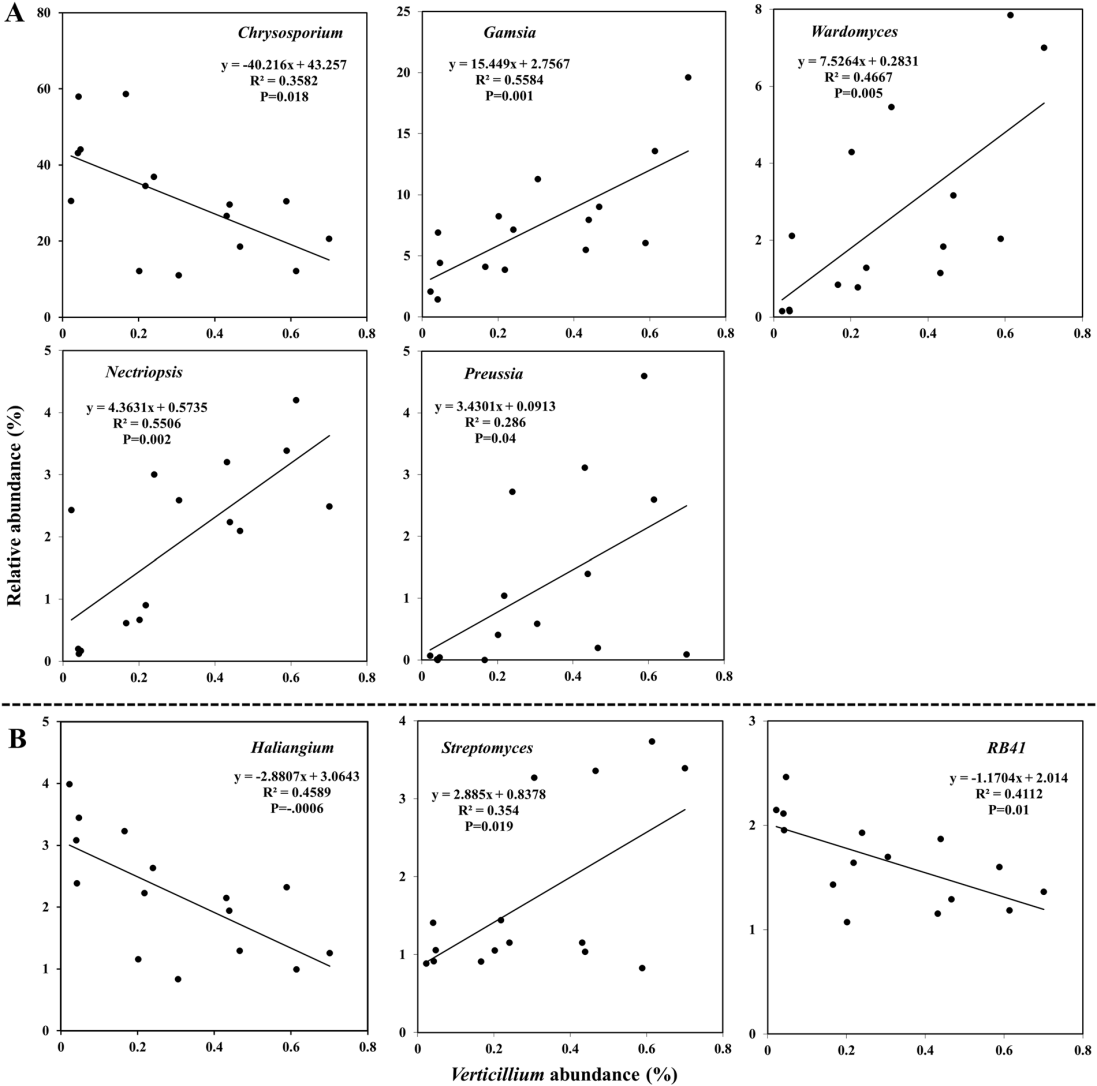
Correlations between abundances of *Verticillium* and other fungal (**A**) or bacterial (**B**) genera. The genera selected with the standard of average relative abundance > 1% over 15 samples and P < 0.05.

5 fungal genera and 17 bacterial genera were found at an average relative abundance > 1%. To determine which genera were closely associated to *Verticillium*, correlation analysis with other genera abundance was conducted. In total, 5 fungal and 6 bacterial genera showed significant correlations to *Verticillium* (Fig. 7). Considering fungi, *Verticillium* was significantly and positively correlated with the abundance of *Chrysosporium*, and negatively correlated with *Gamsia*, *Wardomyces*, *Nectriopsis* and *Preussia* (Fig. 7A). It was positively correlated with *Streptomyces*, but significantly and negatively correlated to *Haliangium* and *RB41*, among bacteria (Fig. 7B).

## Discussion

Verticillium wilt is widespread in many cotton-growing areas, and is the most important cotton disease in China^21^. The current cultivation system (such as long-term continuous cropping, straw returning to the field, drip irrigation, etc.) is very conducive for the occurrence of Verticillium wilt in Xinjiang. In this study, all the tested isolates belonged to the sclerotium type (Fig. S2), with defoliating and nondefoliating pathotypes accounting for 66.7% and 33.3%, respectively. A higher pathogenicity was found for the defoliating isolates, with an average disease index of 32.3, whereas the nondefoliating ones showed an average disease index of 22.6. The strong, moderate and weak pathogenicity isolates accounted for 33.33%, 53.33% and 13.33%, respectively. The results confirmed the importance of Verticillium wilt in Xinjiang, likely related to the high incidence of more and more defoliating and strong pathogenicity isolates. The cultural characteristics and pathogenicity of 15 isolates were significantly different, even when the isolates proceeded from the same cotton field (Table. S1). Some previous studies revealed that there was no correlation between isolate properties and pathogenicity^22–24^. However, we found that spore production was significantly and positively correlated with pathogenicity (Fig. S4).

Data on the relationship between *Verticillium* species and belowground microbiome under long-term continuous cotton cropping system appear important to understand the disease ecology. Due to the limited arable land area in Xinjiang, continuous cotton cropping is a common cultivation pattern. Besides, cotton is usually grown on a large scale in the region. Therefore, it was difficult to find an appropriate field to be used as comparison, nearby the selected cotton fields. Although cotton fields have similar fertilization and tillage patterns across the three regions, both fungal and bacterial soil communities showed differences in alpha- diversity, relative abundance, structure and taxonomic composition, as shown by Venn (Fig. 3B,C) and PCoA (Fig. 4A,B) analysis. The main reason for these differences may likely be related to the different cotton varieties used and to soil properties.

Microbial groups from the same habitat appeared similar, despite proceeding from different sampling sites. For fungi, Ascomycota was the most abundant fungal phylum under continuous cotton cropping, suggesting an enrichment and ubiquity in the cotton monoculture soil ecosystem, consistent with the results of previous studies carried out on soybean^25^, peanut^26^, and vanilla^27^ under continuous cropping regimes. Proteobacteria, Acitnobacteria, Chloroflexi, Acidobacteria and Gemmatimonadetes were the most common bacterial phyla in continuous cotton cropping soils (Fig. 3A). Proteobacteria abundance was highest agreeing with data from several previous studies^16,27–30^, with an important role in the global cycles of carbon, iron, nitrogen and sulphur^31–34^. Actinobacteria take part in the global carbon cycle^35^ and break down soil organic matter^36^. Thus, members of both phyla in continuous cotton cropping soils may have a role in the homeostasis of the soil microbiome.

Many soil properties may have affected microbial communities, according to results (Table 3). *Verticillium* abundance was negatively correlated with OM, AK and pH, and positively correlated only with AN (Fig. 6). Previous studies also revealed the correlations between OM and *Fusarium* abundance^2,37^, pH and tobacco disease rate^16^. Farmers in China applied large amounts of commercial nitrogen and phosphatic fertilizers to achieve higher yields, neglecting the role of organic matter and potash fertilizer for ages^20^. Excessive N and P fertilizers may be a disadvantage for plant growth^38,39^. A variety of soil factors exhibit potential synergistic effects and eventually influence the soil microbial community^40^, which probably underpin the *Verticillium* enrichment in soils.

Moreover, five fungal and six bacterial genera were found significantly correlated with the abundance of *Verticillium* (Fig. 7). The average relative abundance of these related fungal genera in total was 44.11%, much higher than those of the bacterial genera (16.18%), suggesting an important role of fungal communities in keeping *V*. *dahliae* infection. Furthermore, results also suggest that long-term continuous cotton cropping regime may affect the whole structure of soil microbiome primarily through abundance and depletion of certain fungal and bacterial taxa.

Besides the fungal pathogenic *Verticillium*, another soilborne pathogen *Fusarium* was detected. The introduced cotton varieties and the intensive cropping history were associated with the occurrence of Verticillium and Fusarium wilt in the cotton fields^41^. Although the average abundance of *Fusarium* (5.99%) was higher than that of *Verticillium* (0.30%), it showed no significant difference among the three fields. No evident Fusarium wilt could be found when we collected samples, which may due to the popular application of Fusarium wilt-resistant cotton cultivars.

## Conclusion

In this study, we ascertained the cultural characteristics and pathogenicity differentiation of *Verticillium dahliae* in three cotton fields, from three regions in north Xinjiang. The results showed that sclerotium type, defoliating pathotype and moderate pathogenicity isolates were predominated in long-term continuous cotton cropping fields. The isolates showed pathogenicity differentiation, with spore production linked to pathogenicity. Meanwhile, the composition of fungal and bacterial taxa showed significant differences among the three cotton fields. *Verticillium* abundance was negatively correlated with organic matter, available potassium and pH, and positively correlated only with alkali-hydrolyzable nitrogen. *Verticillium* was also positively correlated with abundances of *Chrysosporium* and *Streptomyces*, and negatively correlated with fungi such as *Gamsia*, *Wardomyces*, *Nectriopsis* and *Preussia*, and bacteria such as *Haliangium*, *RB41* and other 3 unidentified genera. These correlations suggested that fungal and bacterial communities around the cotton rhizosphere exhibited similar responses to long-term continuous cropping in north Xinjiang.

## Materials and Methods

### Sample collection and processing

All samples used in this study were collected from cotton fields in north Xinjiang on July 2017 (at the boll stage). One field only was chosen for each of the three main cotton producing cities, Bole (5F, N 44°25′57.035″; E 84°55′26.676″), Kuitun (7F, N 44°20′3.20″; E 86°03′31.85″), and Shihezi (8F, N 44°52′33.326″; E 82°08′43.828″), which have a long-term continuous cotton cropping history and show serious Verticillium wilt disease incidence.

The five *V*. *dahliae* isolates were randomly collected from five diseased cotton plants showing severe Verticillium wilt symptoms in each field. Single spore suspensions were collected and stored in 20% glycerol solution at −80 °C. Meanwhile, five bulk soil samples (15–20 cm in depth) around the roots of diseased cotton plants at the same sites were collected from each field, then put in separate plastic bags. A part of each soil sample was stored at −80 °C for subsequent DNA extraction, the other part being used for physicochemical analysis after air-drying according to previous methods^27,42^.

### Extraction of fungal DNA and pathotype identification

Single spore isolates were grown on potato dextrose agar (PDA) plates for 3 days at 25 °C and then transferred into 20 mL Czapek Dox liquid medium. The mycelia were collected after 3 days to extract DNA using CTAB^43,44^. The DNA quality and concentration were assessed with 1% agarose gel electrophoresis and NANODROP 2000 spectrophotometer (Thermo SCIENTIFIC, USA). The pathotype was checked by PCR assays using two primer pairs, INTD2f (5′-ACTGGGTATGGATGGCTTTCAGGACT-3′)/INTD2r (5′-TCTCGACTATTGGAAAATCCAGCGAC-3′) and INTND2f (5′-CTCTTCGTACATGGCCATAGATGTGC-3′)/INTND2r (5′-CAATGACAATGTCCTGGGTGTGCCA-3′)^45^. PCR procedure: 95 °C for 4 min, followed by 30 cycles of 95 °C for 1 min, 60 °C for 1 min, 72 °C for 30 s, and a final extension of 72 °C for 5 min.

### Cultural characteristics and pathogenicity of *V*. *dahliae*

Isolates V991 and 1cd3-2, previously characterized as defoliating (D) and nondefoliating (ND)^46,47^, were used as reference isolates, respectively. The growth of fungi was monitored by inoculated a plug (0.8 cm diameter) in the centre of PDA plate at 25 °C, measuring the colony diameters at 10 dpi, then photographed at 15 dpi. Each strain consisted of 5 replicates. To estimate conidia production, each isolate was incubated in 20 mL Bilay’s medium with 100 μL of a 2 × 10^5^ spores/mL spore suspension, and incubated on a shaker (180 rpm/min) at 25 °C for 3 days^48,49^, in three replicates. The conidia number were counted under light microscope (BX52, Olympus, Tokyo, Japan).

Two differential hosts were used for pathogenicity assays in a greenhouse at 25 °C, with a 16 h light/8 h dark period. One resistant cultivar, Zhongzhimian 2 and a susceptible one, Xinluzao 36, were used. For each isolate, 30 cotton seedlings were inoculated, by immersing in 10 mL 1 × 10^7^ spores/mL conidial suspension for each seedling. Sterile distilled water was used as control. Disease index (DI) was used to evaluate the severity of Verticillium wilt scaling from 0 to 4^50^, using the following formula: DI = [Σ (disease grades × number of infected plants)/(total checked plants × 4)] × 100^51^. This experiment repeated twice, the data were presented as means ± standard deviation, significance difference was calculated by SPSS (v.17.0).

### Soil DNA extraction, PCR amplification and illumina MiSeq sequencing

Total soil DNA was extracted from 15 samples using the DNeasy PowerSoil Kit (QIAGEN, Germany) according to the instructions. The fungi-specific primers pairs: ITS1F (5′-CTTGGTCATTTAGAGGAAGTAA-3′)^52^ and ITS2 (5′-GCTGCGTTCTTCATCGATGC-3′)^53^ were used to amplify the ITS1 region. The bacteria-specific primer pairs: 341F (5′-CCTACGGGNGGCWGCAG-3′) and 805 R (5′-GACTACHVGGGTATCTAATCC-3′) were used to amplify the V3-V4 hypervariable region of 16S rRNA gene^18^. PCR reaction system and program were performed following a previous study^27^. PCR products were purified with Gel Extraction Kit (OMEGA, USA) and pooled in equimolar concentrations. Then, paired-end sequencing of fungal and bacterial amplicons was carried out on an Illumina MiSeq platform at Personal Biotechnology Co., Ltd (Shanghai, China)^54^.

### Statistical analyses

After discarding the no-target amplicon sequence variants (ASVs) and low- abundance ASVs (<5 total counts)^55^, the dataset was normalized to the minimum number of read counts, and the analyses of alpha and beta diversity were performed with QIIME 2^56^. Alpha-diversity analyses included Shannon, Chao1, richness, evenness, phylogenetic diversity and coverage, calculated using Mothur (v.1.34.4)^57^. Beta diversity was calculated by weighted UniFrac distance and analyzed by Principal coordinate analyses (PCoA)^29^. The Kruskal-Wallis test and permutational multivariate analysis of variance (PERMANOVA) with 999 random permutations were respectively used to analyze statistical differences in alpha-diversity and beta diversity^58^. Venn diagrams were implemented online to show unique and shared OTUs (Operational Taxonomic Units) (http://bioinformatics.psb.ugent.be/webtools/Venn/). Correlations between soil chemical properties and soil microbiome community were calculated by Mantel tests to construct dissimilarity matrices using R package (v. 3.5.3) via Bray-Curtis and Euclidean distance for bacterial and fungal community, respectively^59^. One-way analysis of variance with Turkey-Kramer test were used for the multiple comparison analyses using STAMP (v.2.0.0)^60–62^. The differences of growth rate, spore production, disease index and soil chemical properties were determined by one-way analysis of variance (ANOVA) or t tests in SPSS (v.17.0). Pearson correlations coefficients was used to test the correlation significance between spore production and disease index, or soil chemical properties and alpha-diversity indices or *Verticillium* abundance, or fungal/bacterial community and *Verticillium* abundance.

## Supplementary information

Supplementary Materials
