# Supplementary material for: Effects of Long-term Cotton Continuous Cropping on Soil Microbiome
Source: Sci Rep. 2019 Dec 4;9:18297. doi: 10.1038/s41598-019-54771-1 (PMC6892916; doi:10.1038/s41598-019-54771-1)
Supplement: Supplementary file 1 — Supplementary Materials [file 41598_2019_54771_MOESM1_ESM.pdf]

Supporting information

Effects of Long-term Cotton Continuous Cropping on Soil Microbiome

Hui Xi[1], Jili Shen[1], Zheng Qu[2], Dingyi Yang[3], Shiming Liu[3], Xinhui Nie[1] & Longfu Zhu[3*]

[1] College of Agronomy, Shihezi University, Shihezi 832000, P.R. China.

[2] State Key Laboratory of Agricultural Microbiology, Huazhong Agricultural University, Wuhan 430070, Hubei, P.R. China.

[3] National Key Laboratory of Crop Genetic Improvement, Huazhong Agricultural University, Wuhan 430070, Hubei, P.R. China.

* To whom correspondence should be addressed.

National Key Laboratory of Crop Genetic Improvement, Huazhong Agricultural University, Wuhan, Hubei 430070, China

Tel: +86-2787283955

E-mails: lfzhu@mail.hzau.edu.cn

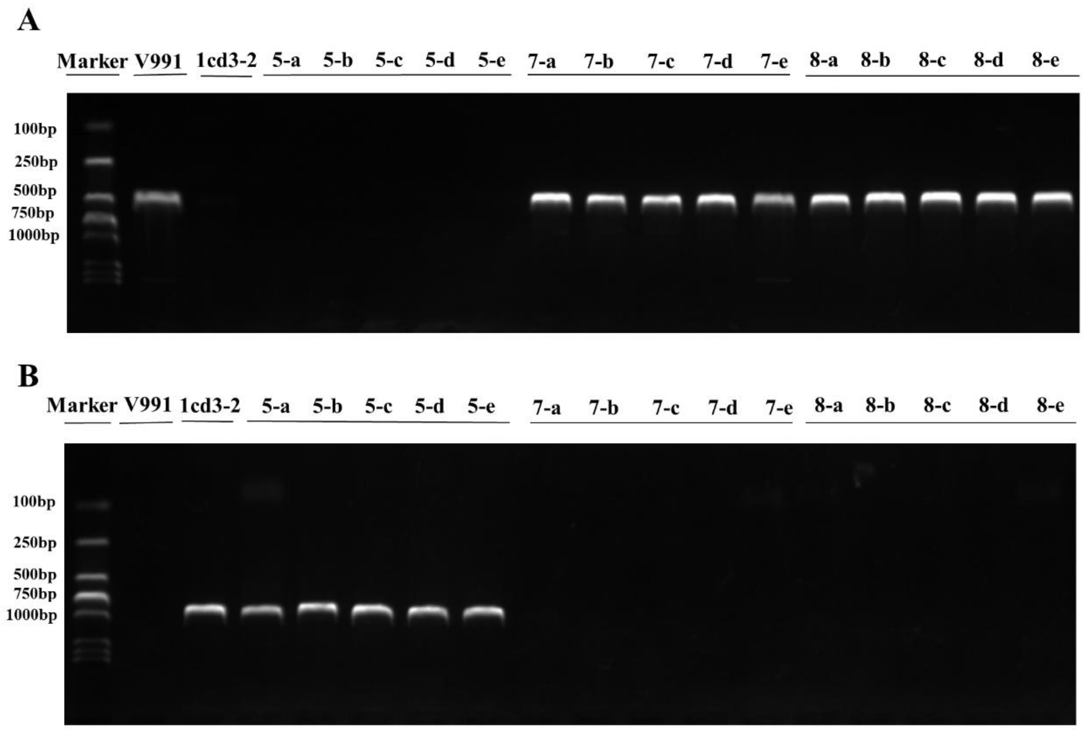

Figure S1. Identification of the defoliating (A) and non-defoliating (B) pathotypes of all isolates with the corresponding molecular marker (Full-length gels).

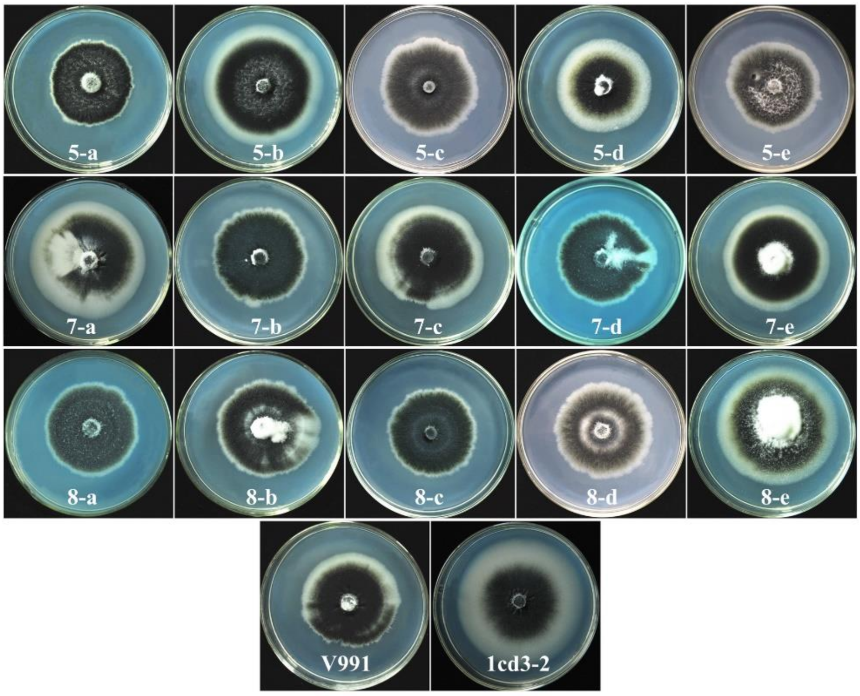

Figure S2. Colony morphology of 15 isolates and 2 reference isolates.

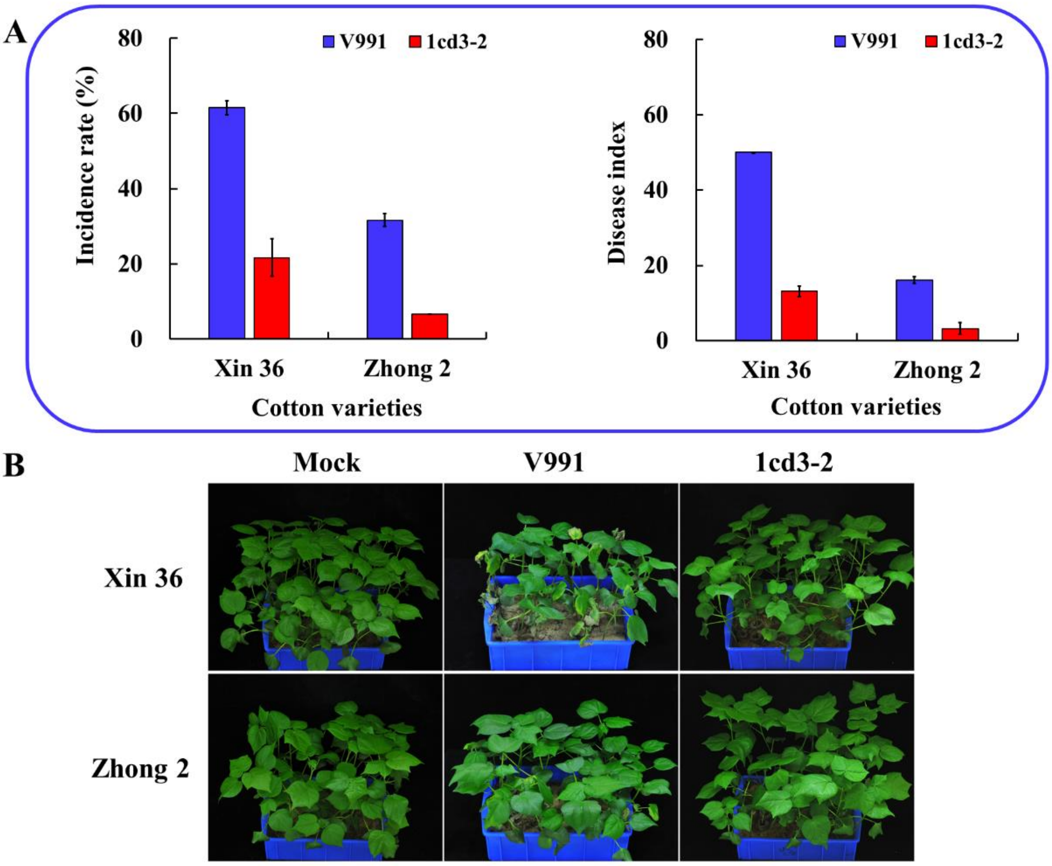

Figure S3. The disease incidence and phenotype of two cotton varieties after inoculated with reference isolates.

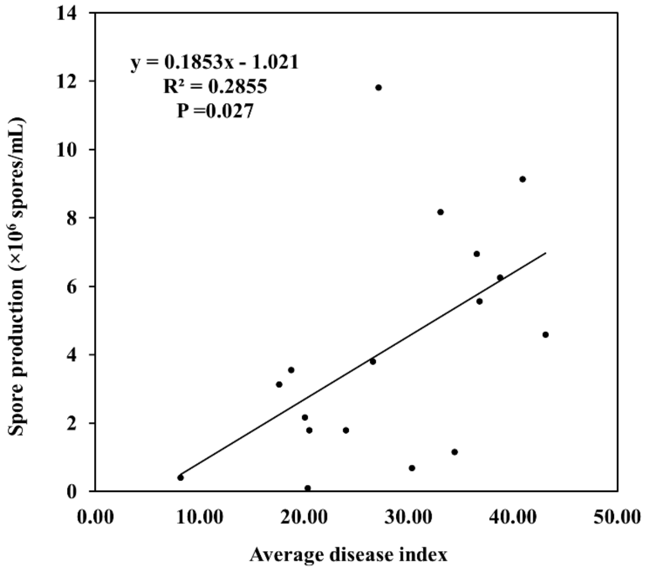

Figure S4. The correlations between spore production and average disease index.

Isolates	Growth rate (cm/d)	Spore production (× 10^6 spores/mL)	Disease index		Average disease index
			Xinluzao 36	Zhongzhimian 2	
V991	0.19± 0.02cd	8.17± 0.80abc	50.00± 0.00abcd	16.14± 0.82bcde	33.07± 0.41abcde
1cd3-2	0.25± 0.00a	0.4± 0.18g	13.10± 1.42e	3.26± 1.59f	8.17± 1.51g
5-a	0.20± 0.01bcd	0.10± 0.00g	33.28± 4.25de	7.37± 0.70ef	20.32± 1.78defg
5-b	0.20± 0.01bcd	1.78± 1.38efg	27.08± 9.22de	13.92± 1.41cde	20.49± 5.32defg
5-c	0.19± 0.01cd	1.78± 1.53efg	37.44± 7.60cde	10.58± 4.74def	24.01± 1.43cdef
5-d	0.19± 0.00cd	3.13± 1.95defg	24.98± 1.63de	10.23± 1.06def	17.61± 1.35fg
5-e	0.19± 0.00cd	0.68± 0.12fg	41.73± 1.02bcde	18.94± 4.45abcd	30.33± 2.73abcdef
7-a	0.20± 0.00bcd	11.82± 0.28a	34.44± 0.57de	19.79± 1.24abc	27.11± 0.9bcdef
7-b	0.18± 0.01cd	3.80± 1.50defg	42.48± 5.38bcde	10.66± 0.18def	26.57± 2.78bcdef
7-c	0.19± 0.01cd	6.25± 3.02bcd	64.46± 19.42abc	13.04± 4.70cde	38.75± 12.06abc
7-d	0.20± 0.01bcd	3.55± 0.76defg	26.89± 3.50de	10.65± 0.64def	18.77± 1.43efg
7-e	0.18± 0.01cd	2.17± 1.52efg	30.64± 0.80de	9.47± 1.41ef	20.05± 1.10defg
8-a	0.20± 0.00bcd	5.57± 0.67bcde	48.62± 10.26abcd	24.95± 3.27a	36.79± 6.77abc
8-b	0.18± 0.01cd	9.13± 0.93ab	68.24± 19.67ab	13.58± 3.10cde	40.90± 8.29ab
8-c	0.21± 0.01bc	4.58± 0.16cdef	71.93± 13.55a	14.30± 3.82cde	43.12± 4.87a
8-d	0.17± 0.01d	6.95± 1.27bcd	50.03± 0.84abcd	23.01± 2.85ab	36.52± 1.84abc
8-e	0.22± 0.00b	1.15± 0.12fg	42.18± 2.18bcde	26.67± 0.86a	34.42± 0.66abcd

Table S1. Cultural characteristics of *V. dahliae* isolates and the results of pathogenicity on two cotton varieties. Isolates from field 5F including 5-a, 5-b, 5-c, 5-d and 5-e; isolates from field 7F including 7-a, 7-b, 7-c, 7-d and 7-e; isolates from field 8F including 8-a, 8-b, 8-c, 8-d and 8-e. (Values are means ± standard deviation. The followed same letter for a given factor are not significantly difference. $P < 0.05$.)
